# Current News

**Published:** 1889-11

**Authors:** 


### Current News.

FIRST DISTRICT DENTAL SOCIETY, New York, January 14, 15, 16, 1890. Keep the date in mind, and your appointment-book free.

Dr. J. N. Crouse will, in all probability, be there, and may want to "see" you, as the civil suit against him is set for some time about the first of the year.

At any rate, you had better "see" Dr. Crouse, in order to protect yourself against civil suits or injunctions. Remember it only costs ten dollars to get a guarantee against all costs in case you are sued.

---

To rotate a tooth, Dr. R. B. Adair has a platinum band, to which is soldered a little cylinder; one end of a coil of very fine piano-wire is placed in the cylinder; the other is flattened and passed between the teeth. Almost any tooth can be thus rotated in two or three days with very little soreness or annoyance.

---

DR. H. S. COLDING holds that dentists are born, not made by colleges. He also thinks that the practice of dentistry is two-thirds mechanics and one-third common sense, medicine, and surgery.

Predisposing Causes.—Nationality has a supposed predisposing influence, but statistics are not sufficiently accurate to warrant definite conclusions upon this point. Heredity has its undoubted effects, particularly in determining the forms and arrangements of the teeth, and, in a more ill-defined way, of transmitting from parent to child the peculiar character of cellular elements, rendering the child susceptible to disease. To malformation of individual teeth is directly traceable one of the causes predisposing to caries.

W. C. Wardlaw.

---

At the Georgia State Meeting, Dr. S. A. White reported the case of a child brought to him suffering from a very sore nose, with a very painful protuberance in one nostril. Examining the teeth, he found the right lateral and the left central incisor touching, with no trace of the right central and no room for it. The teeth being separated, in a short time the soreness of the nose diminished, the protuberance disappeared, and the missing central incisor came down all right into place.

---

In the treatment of chronic alveolar abscess, Dr. R. B. Adair uses a delicate bulb made of platinum and slightly curved at the end, so as to follow the track; the point is heated to red heat, and pressed on a stick of nitrate of silver, which adheres to the point and hardens; this is carried to the bottom of the abscess, and turned around so as to cauterize the edge of necrosed bone, destroy micro-organisms, and stimulate to healthy action.

---

President S. A. White, of the Georgia State Dental Society, thinks it the duty of the State Society to help the young men, especially by making them members of the Society. Their college expenses have been heavy; fitting up an office is a heavy expense; travelling expenses to meet the State Examining Board must be met; their license fee must be paid,—all before they can honestly earn their first dollar professionally.

---

Instead of a plate covering the roof of the mouth, Dr. S. A. White sets the teeth on a gold band or wire one-fourth of an inch wide, making a skeleton-plate which is very strong and worn with absolute comfort.

Dr. B. H. Catching thinks the admission to their State Society of young men fresh from college should not be made a matter of dollars and cents. They are the material with which to build up the profession in the State, and, adopted from the start with their State Society, they will grow up with its best interests at heart.

---

Dr. Sid Holland thinks that a young man who has not sufficient natural ambition to pay his own initiation-fee into his State Society is not worth "fostering." He is not made of the right material to build up a society with.

---

Dr. Geo. H. Winkler finds that bichloride of mercury forced through the apex gives very severe toothache.

---

Notice.—The First District Dental Society of the State of New York will hold its twenty-first anniversary in New York City, January 14, 15, and 16, 1890, on which occasion every practising dentist will be cordially invited. Special railroad and hotel rates will be made. Please note date of meeting, and make your appointments accordingly.

All communications should be addressed to the Executive Committee.

W. W. Walker, *Chairman*,
67 West Ninth Street, New York City.

---

Pennsylvania Association of Dental Surgeons.—At the annual meeting of this Society the following officers were elected to serve for the ensuing year: Howard E. Roberts, President; C. F. Bonsall, Vice-President; Theodore F. Chupein, Recording and Corresponding Secretary and Reporter; W. H. Trueman, Treasurer and Librarian.

---

Maryland State Dental Association.—The sixth annual meeting of the Maryland State Dental Association will be held in Baltimore on Thursday and Friday, December 5 and 6. The programme will consist of reports from committees, essays, clinics, and displays of dental specialties.

D. F. Penington, M.D., *Recording Secretary.*
E. P. Keech, M.D., D.D.S., *President.*

